# Supplementary material for: PARP10 promotes the repair of nascent strand DNA gaps through RAD18 mediated translesion synthesis
Source: Nat Commun. 2024 Jul 23;15:6197. doi: 10.1038/s41467-024-50429-3 (PMC11266678; doi:10.1038/s41467-024-50429-3)
Supplement: Supplementary file 1 — Supplementary Information [file 41467_2024_50429_MOESM1_ESM.pdf]

Supplementary Information

PARP10 promotes the repair of nascent strand DNA gaps through RAD18 mediated Translesion Synthesis

Khatib, Dhoonmoon, Moldovan and Nicolae

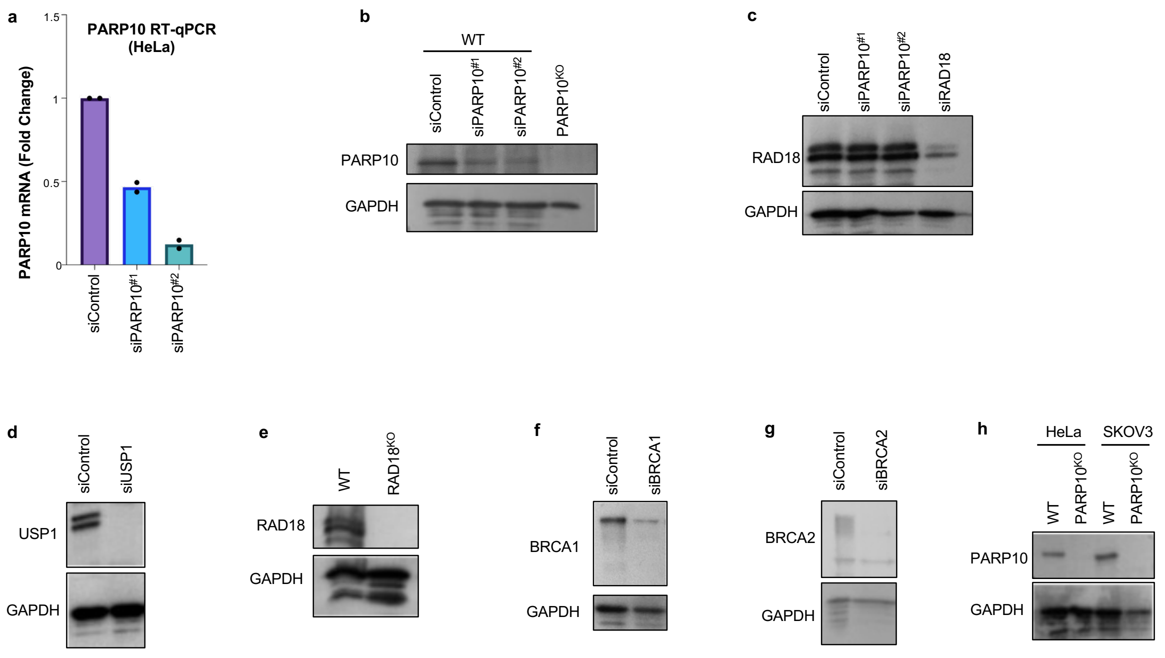

Supplementary Figure 1. Confirmation of gene knockdowns and knockouts.

a. RT-qPCR experiment showing siRNA-mediated knockdown of PARP10. The average of two technical replicates is shown. Total mRNA was purified using TRIzol reagent (Life Tech). To generate cDNA, 1μg RNA was subjected to reverse transcription using the RevertAid Reverse Transcriptase Kit (Thermo Fisher Scientific) with oligo-dT primers. Real-time qPCR was performed with PerfeCTa SYBR Green SuperMix (Quanta), using a CFX Connect Real-Time Cycler (BioRad). The cDNA of GAPDH gene was used for normalization. Primers used were: GAPDH for: AAATCAAGTGGGGCGATGCTG; GAPDH rev: GCAGAGATGATGACCCTTTTG; PARP10 for: CTGTGGACCTGCTGTTGCTG; PARP10 rev: GGATGTCGTAGTGGGGGACA.

b-f. Western blots showing the siRNA-mediated knockdown of PARP10 (**b**), RAD18 (**c**), USP1 (**d**), BRCA1 (**f**) and BRCA2 (**g**) in HeLa cells, the CRISPR/Cas9-mediated knockout of PARP10 in HeLa (**b,h**) and SKOV3 (**g**) cells, as well as the CRISPR/Cas9-mediated knockout of RAD18 in HeLa cells (**e**).

Source data are provided as a Source Data file.

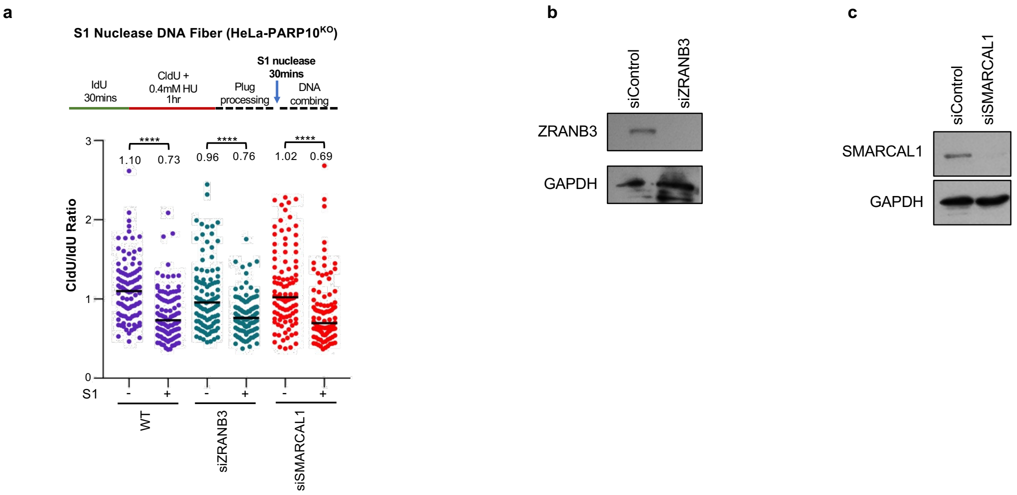

Supplementary Figure 2. **Impact of fork reversal on PARP10-mediated gap suppression.**

a. S1 nuclease DNA fiber combing assays showing that depletion of fork reversal translocases ZRANB3 and SMARCAL1 does not affect the accumulation of ssDNA gaps induced by treatment with 0.4mM HU in PARP10-knockout HeLa cells. The ratio of CldU to IdU tract lengths is presented, with the median values marked on the graphs and listed at the top. At least 100 tracts were quantified for each sample. Asterisks indicate statistical significance (Mann-Whitney, two-tailed). A schematic representation of the assay conditions is shown at the top.

b,c. Western blots showing the siRNA-mediated knockdown of ZRANB3 (**b**) and SMARCAL1 (**c**).

Source data are provided as a Source Data file.

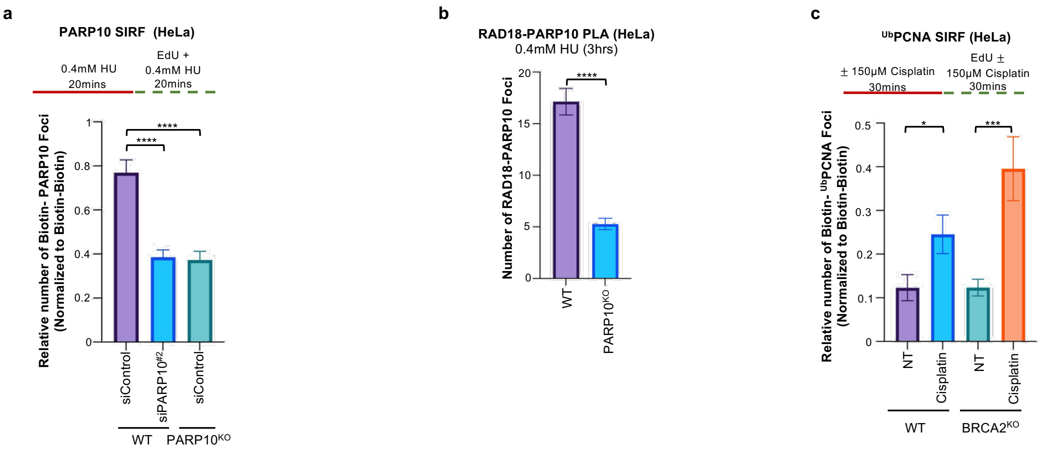

Supplementary Figure 3. **Control PLA experiments.**

a. SIRF experiments showing the binding of PARP10 to nascent DNA upon treatment with 0.4mM HU in HeLa cells. PARP10 depletion and PARP10 knockout are used as controls. At least 85 cells were quantified for each condition. Bars indicate the mean values, error bars represent standard errors of the mean, and asterisks indicate statistical significance (t-test, two-tailed, unpaired). Schematic representations of the assay conditions are shown at the top.

b. PLA assay showing the co-localization between RAD18 and PARP10 upon treatment with 0.4mM HU for 3hrs in HeLa cells. PARP10 knockout is used as control to confirm the specificity of the PLA signals observed. At least 76 cells were quantified for each condition. Bars indicate the mean values, error bars represent standard errors of the mean, and asterisks indicate statistical significance (t-test, two-tailed, unpaired).

c. SIRF experiments showing the levels of ubiquitinated PCNA on nascent DNA. At least 50 cells were quantified for each condition. Bars indicate the mean values, error bars represent standard errors of the mean, and asterisks indicate statistical significance (t-test, two-tailed, unpaired). A schematic representation of the assay conditions is shown at the top.

Source data are provided as a Source Data file.

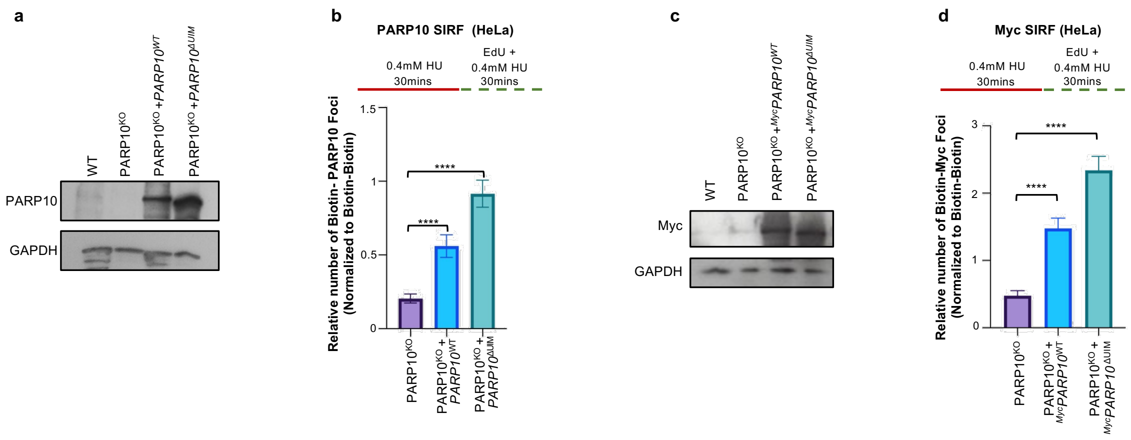

Supplementary Figure 4. **Impact of PARP10 UIM motifs on its localization to nascent DNA.**

a. Western blots showing the exogenous re-expression of wildtype and ΔUIM PARP10 variants in PARP10-knockout HeLa cells.

b. PARP10 SIRF experiment showing the impact of the UIM motifs deletion on PARP10 localization to nascent DNA upon exposure to 0.4mM HU. At least 58 cells were quantified for each condition. Bars indicate the mean values, error bars represent standard errors of the mean, and asterisks indicate statistical significance (t-test, two-tailed, unpaired). A schematic representation of the assay conditions is shown at the top.

c,d. These findings were confirmed using transient expression of Myc-tagged PARP10 and $PARP10^{\Delta UIM}$ in PARP10-knockout HeLa cells (**c**), using CMV-Myc-PARP10 constructs we previously described (ref. 40) and Myc SIRF experiments (**d**). At least 60 cells were quantified for each condition. Bars indicate the mean values, error bars represent standard errors of the mean, and asterisks indicate statistical significance (t-test, two-tailed, unpaired). A schematic representation of the assay conditions is shown at the top.

Source data are provided as a Source Data file.

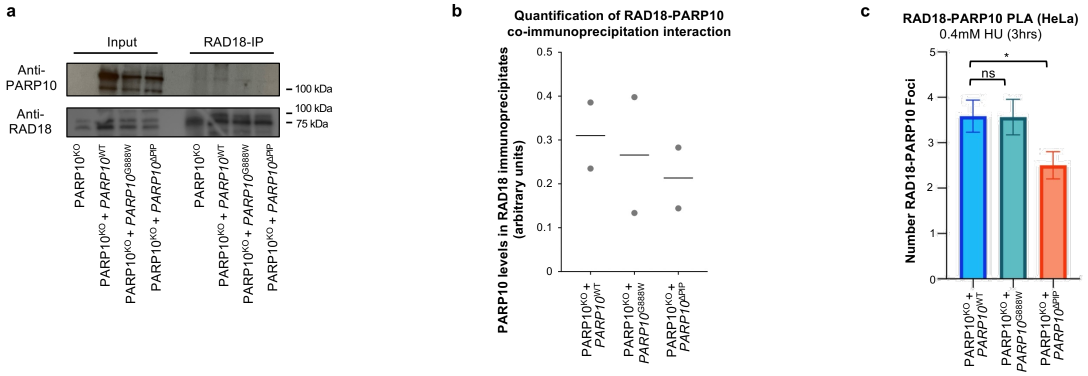

Supplementary Figure 5. **Interaction of PARP10 variants with RAD18.**

a. Co-immunoprecipitation experiment in HeLa cells showing the interaction of PARP10 variants with RAD18.

b. Quantification of two RAD18-PARP10 co-immunoprecipitation experiments (shown in Fig. 5j and Supplementary Figure 5a), using ImageJ.JS v0.5.8 (https://ij.imjoy.io). Shown are PARP10 levels in RAD18 immuno-precipitates, normalized to Input levels. The line indicates median values.

c. PLA assay showing the co-localization between RAD18 and PARP10 variants upon treatment with 0.4mM HU for 3hrs in HeLa cells. The catalytic site mutant (G888W) shows similar RAD18 co-localization as the wildtype form, while the PCNA interaction-deficient (∆PIP) mutant shows reduced RAD18 co-localization. At least 50 cells were quantified for each condition. Bars indicate the mean values, error bars represent standard errors of the mean, and asterisks indicate statistical significance (t-test, two-tailed, unpaired).

Source data are provided as a Source Data file.

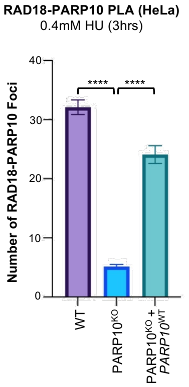

Supplementary Figure 6. **Control PLA experiments showing the re-expression of PARP10 in PARP10-knockout cells.**

PLA assay showing the co-localization between RAD18 and PARP10 upon treatment with 0.4mM HU for 3hrs in HeLa cells. PARP10 knockout is used as control to confirm the specificity of the PLA signals observed. At least 52 cells were quantified for each condition. Bars indicate the mean values, error bars represent standard errors of the mean, and asterisks indicate statistical significance (t-test, two-tailed, unpaired).

Source data are provided as a Source Data file.

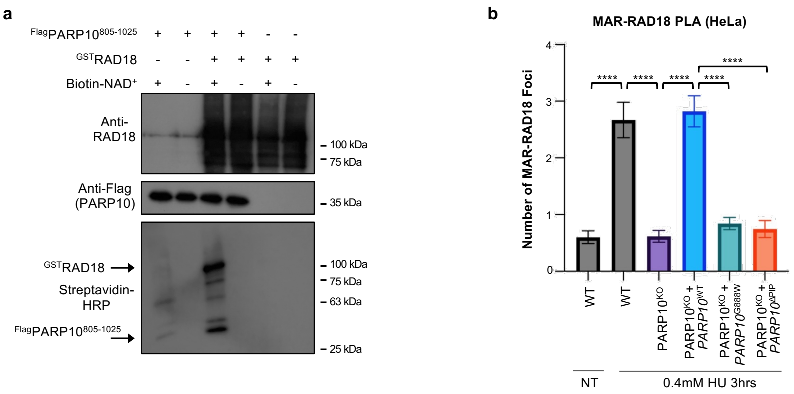

Supplementary Figure 7. **RAD18 mono-ADP-ribosylation by PARP10.**

a. *In vitro* ADP-ribosylation enzymatic assays showing that PARP10 can MARylate RAD18. Recombinant proteins were incubated with biotin-NAD^+ and substrate MARylation is detected using streptavidin-HRP blots. RAD18 and Flag (PARP10) blots are presented to show loading controls.

b. PLA assay showing the impact of PARP10 mutations on the co-localization between RAD18 and mono-ADP-ribose. Expression of wildtype PARP10, but not of the G888W catalytic site mutant or the PIP-box mutant restored the MAR-RAD18 PLA signal in $PARP10^{KO}$ cells. At least 50 cells were quantified for each condition. Bars indicate the mean values, error bars represent standard errors of the mean, and asterisks indicate statistical significance (t-test, two-tailed, unpaired).

Source data are provided as a Source Data file.

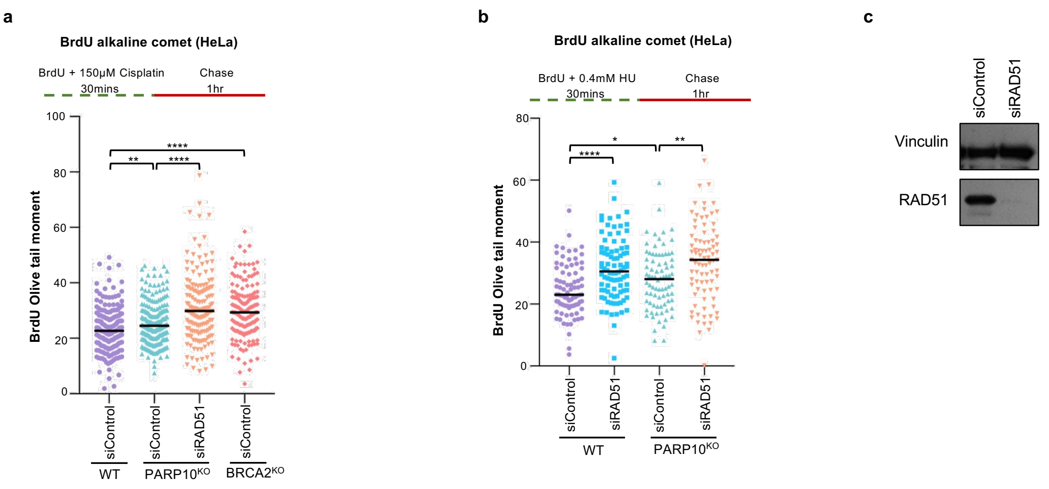

Supplementary Figure 8. **RAD51 depletion enhances ssDNA gap accumulation in PARP10-deficient cells.**

a,b. BrdU alkaline comet assays showing that loss of RAD51 enhances the accumulation of replication-associated ssDNA gaps upon treatment with 150μM cisplatin (**a**) or 0.4mM HU (**b**) in HeLa PARP10-knockout cells. At least 80 nuclei were quantified for each condition. The median values are marked on the graph and listed at the top. Asterisks indicate statistical significance (Mann-Whitney, two-tailed). Schematic representations of the assay conditions are shown at the top.

c. Western blots showing the siRNA-mediated knockdown of RAD51.

Source data are provided as a Source Data file.

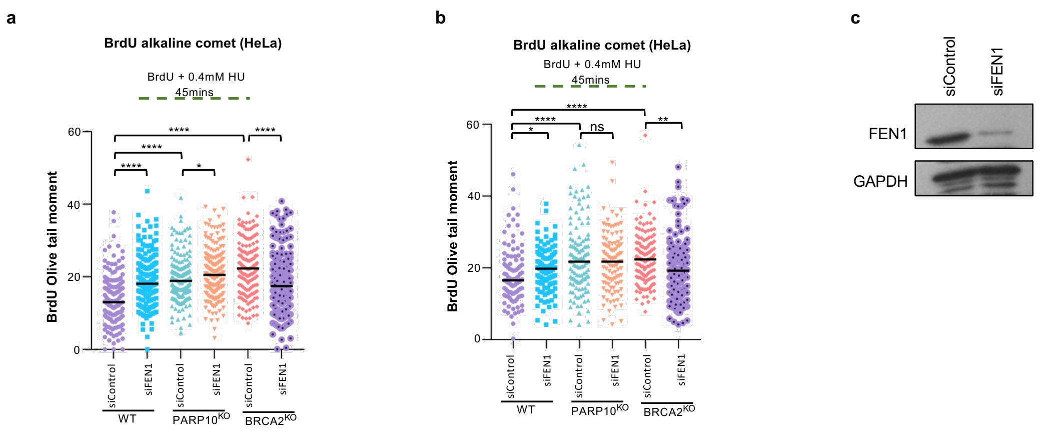

Supplementary Figure 9. **FEN1 depletion does not impact ssDNA gap accumulation in PARP10-deficient cells.**

a,b. BrdU alkaline comet assays showing that loss of FEN1 suppresses the accumulation of replication-associated ssDNA gaps upon treatment with 0.4mM HU in HeLa-BRCA2^{KO} cells, but not in PARP10^{KO} cells. At least 100 nuclei were quantified for each condition. The median values are marked on the graph and listed at the top. Asterisks indicate statistical significance (Mann-Whitney, two-tailed). A schematic representation of the assay conditions are shown at the top.

c. Western blots showing the siRNA-mediated knockdown of FEN1.

Source data are provided as a Source Data file.

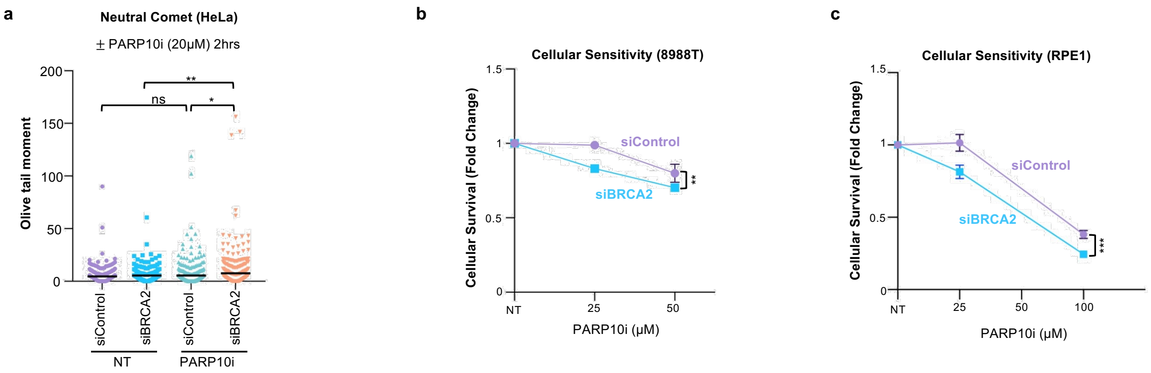

Supplementary Figure 10. Impact of PARP10 inhibitors on the proliferation of BRCA2-depleted cells.

a. Neutral comet assay showing that treatment of BRCA2-knockdown HeLa cells with OUL35 results in increased DSB formation. At least 120 comets were quantified for each sample. The median values are marked on the graph, and asterisks indicate statistical significance (Mann-Whitney, two-tailed).

b,c. Cellular viability assays showing that PARP10 inhibition using the specific inhibitor OUL35 causes cytotoxicity in BRCA2-depleted 8988T (**a**) and RPE1 (**b**) cells. The average of three independent experiments, with standard deviations indicated as error bars, is shown. Asterisks indicate statistical significance (two-way ANOVA).

Source data are provided as a Source Data file.

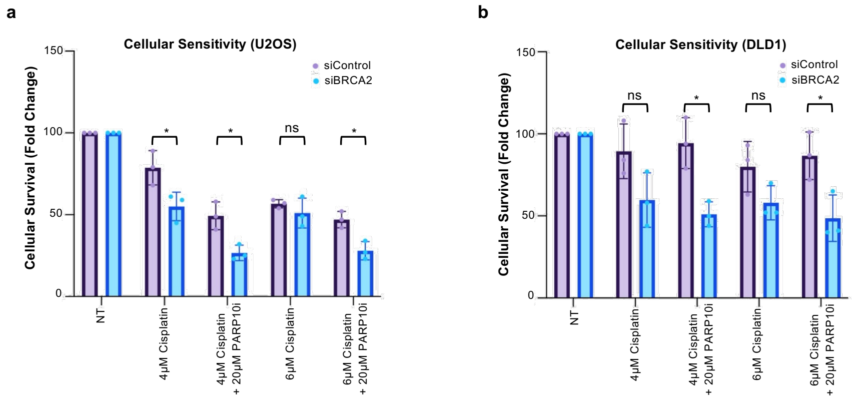

Supplementary Figure 11. **Impact of PARP10 inhibitors on the cisplatin sensitivity of BRCA2-depleted cells.**

Cellular viability assays showing that BRCA2-depleted U2OS (**a**) and DLD1 (**b**) cells have increased sensitivity to co-treatment with cisplatin and OUL35. The average of three independent experiments, with standard deviations indicated as error bars, is shown. Asterisks indicate statistical significance (t-test, two-tailed, unpaired).

Source data are provided as a Source Data file.
