# Supplementary material for: PARP10 promotes the repair of nascent strand DNA gaps through RAD18 mediated translesion synthesis
Source: Nat Commun. 2024 Jul 23;15:6197. doi: 10.1038/s41467-024-50429-3 (PMC11266678; doi:10.1038/s41467-024-50429-3)
Supplement: Supplementary file 3 — Reporting Summary [file 41467_2024_50429_MOESM3_ESM.pdf]

nature portfolio

Corresponding author(s): Claudia M Nicolae

Last updated by author(s): Jun 14, 2024

Reporting Summary

Nature Portfolio wishes to improve the reproducibility of the work that we publish. This form provides structure for consistency and transparency in reporting. For further information on Nature Portfolio policies, see our Editorial Policies and the Editorial Policy Checklist.

Statistics

For all statistical analyses, confirm that the following items are present in the figure legend, table legend, main text, or Methods section.

n/a	Confirmed	
☐	☒	The exact sample size (*n*) for each experimental group/condition, given as a discrete number and unit of measurement
☐	☒	A statement on whether measurements were taken from distinct samples or whether the same sample was measured repeatedly
☐	☒	The statistical test(s) used AND whether they are one- or two-sided<br>*Only common tests should be described solely by name; describe more complex techniques in the Methods section.*
☒	☐	A description of all covariates tested
☒	☐	A description of any assumptions or corrections, such as tests of normality and adjustment for multiple comparisons
☐	☒	A full description of the statistical parameters including central tendency (e.g. means) or other basic estimates (e.g. regression coefficient) AND variation (e.g. standard deviation) or associated estimates of uncertainty (e.g. confidence intervals)
☐	☒	For null hypothesis testing, the test statistic (e.g. *F*, *t*, *r*) with confidence intervals, effect sizes, degrees of freedom and *P* value noted<br>*Give P values as exact values whenever suitable.*
☒	☐	For Bayesian analysis, information on the choice of priors and Markov chain Monte Carlo settings
☒	☐	For hierarchical and complex designs, identification of the appropriate level for tests and full reporting of outcomes
☒	☐	Estimates of effect sizes (e.g. Cohen's *d*, Pearson's *r*), indicating how they were calculated

Our web collection on statistics for biologists contains articles on many of the points above.

Software and code

Policy information about availability of computer code

Data collection	1) DNA Fiber Combing, Immunofluorescence: LASX 3.5.7.23225<br>2) Proximity Ligation Assays (PLA) and In-Situ Analysis of Protein Interactions at DNA Replication Forks (SIRF): SoftWorx 6.5.2<br>3) CometAssay: NIS Elements V1.10.00
Data analysis	1) DNA Fiber Combing: LASX 3.5.7.23225<br>2) In-Situ Analysis of Protein Interactions at DNA Replication Forks (SIRF): ImageJ 1.53a<br>3) Proximity Ligation Assay (PLA): ImageJ 1.53a<br>4) Comet Assay: CometScore 2.0<br>5) Statistics and Data Figure Panel: Graphpad Prism 10 and Microsoft Excel v2205<br>6) Western blot image quantifications: ImageJ.JS v0.5.8 run in browser (https://ij.imjoy.io).

For manuscripts utilizing custom algorithms or software that are central to the research but not yet described in published literature, software must be made available to editors and reviewers. We strongly encourage code deposition in a community repository (e.g. GitHub). See the Nature Portfolio guidelines for submitting code & software for further information.

Data

Policy information about availability of data

All manuscripts must include a data availability statement. This statement should provide the following information, where applicable:

- Accession codes, unique identifiers, or web links for publicly available datasets
- A description of any restrictions on data availability
- For clinical datasets or third party data, please ensure that the statement adheres to our policy

All data presented in the manuscript is provided as a Source Data file included with the manuscript.

Research involving human participants, their data, or biological material

Policy information about studies with human participants or human data. See also policy information about sex, gender (identity/presentation), and sexual orientation and race, ethnicity and racism.

Reporting on sex and gender	Not applicable
Reporting on race, ethnicity, or other socially relevant groupings	Not applicable
Population characteristics	Not applicable
Recruitment	Not applicable
Ethics oversight	Not applicable

Note that full information on the approval of the study protocol must also be provided in the manuscript.

Field-specific reporting

Please select the one below that is the best fit for your research. If you are not sure, read the appropriate sections before making your selection.

[x] Life sciences [ ] Behavioural & social sciences [ ] Ecological, evolutionary & environmental sciences

For a reference copy of the document with all sections, see nature.com/documents/nr-reporting-summary-flat.pdf

Life sciences study design

All studies must disclose on these points even when the disclosure is negative.

Sample size	No statistical method was used to predetermine sample size. Sample size was determined based on previous experiments and relevant literature in the field (eg Taglialatela et al, PMID: 29053959; Lemacon et al, PMID: 29038425; Tirman et al, PMID: 34624216). Statistical significance was obtained with this sample size.
Data exclusions	No data was excluded from the analysis.
Replication	All experiments were performed at least twice. All results were reproducible.
Randomization	Samples were randomly allocated into experimental groups. Samples were processed and analyzed in random order.
Blinding	For imaging-based assays, samples were tested at least once under blinding conditions, and results were reproduced. Blinding was not performed for western blot experiments, since those experiments were simply aimed at validating antibodies and thus blinding was deemed irrelevant.

Reporting for specific materials, systems and methods

We require information from authors about some types of materials, experimental systems and methods used in many studies. Here, indicate whether each material, system or method listed is relevant to your study. If you are not sure if a list item applies to your research, read the appropriate section before selecting a response.

Materials & experimental systems

n/a	Involved in the study
☐	☒ Antibodies
☐	☒ Eukaryotic cell lines
☒	☐ Palaeontology and archaeology
☒	☐ Animals and other organisms
☒	☐ Clinical data
☒	☐ Dual use research of concern
☒	☐ Plants

Methods

n/a	Involved in the study
☒	☐ ChIP-seq
☒	☐ Flow cytometry
☒	☐ MRI-based neuroimaging

Antibodies

Antibodies used

Antibodies used for Western blot, at 1:500 dilution, were:
PARP10: Abcam ab70800;
RAD18: Cell Signaling Technology 9040;
USP1: Abcam ab264221;
BRCA1 Santa Cruz Biotechnology sc-6954;
BRCA2 Bethyl A303-434A;
FEN1: Santa Cruz Biotechnology sc-28355;
SMARCAL1: Invitrogen PA5-54181;
ZRANB3: Invitrogen PA5-6514;
RAD51: Santa Cruz Biotechnology sc-8349;
Streptavidin-HRP: ThermoFisher 21130;
FLAG: Cell Signaling Technology 14793;
Myc: Santa Cruz Biotechnology sc-40;
Vinculin: Santa Cruz Biotechnology sc-73614;
GAPDH: Santa Cruz Biotechnology sc-47724.

Antibodies used for the BrdU alkaline comet assay were: anti-BrdU (BD 347580) and secondary AF568-conjugated antibodies (Invitrogen A-11031).

Antibodies used for DNA fiber combing were: CldU (Abcam 6236) and IdU (BD 347580), and secondary Cy3 (Abcam 6946) and Cy5 (Abcam 6565) conjugated antibodies.

Antibodies used for SIRF assays were Biotin (mouse: Jackson ImmunoResearch 200-002-211; rabbit: Bethyl Laboratories A150-109A); MRE11 (GeneTex GTX70212); PARP10 (Abcam ab70800); RAD18 (Cell Signaling Technology 9040); Ubiquityl-PCNA Lys164 (Cell Signaling Technology 13439); REV1 (Santa Cruz Biotechnology sc-393022); Myc (Santa Cruz Biotechnology sc-40).

Antibodies used for PLA assays were PARP10 (Abcam ab70800); MAR AbD33204 (BioRad HCA354) and RAD18 (Cell Signaling Technology 9040).

Antibodies used for co-immunoprecipitation assays were RAD18 (Cell Signaling Technology 9040) and control rabbit IgG (GenScript A01008).

Validation

All antibodies were validated by western blots and imaging-based SIRF assays using gene inactivation (siRNA-mediated knockdown or CRISPR-mediated knockout) as negative control. The data is presented in the manuscript (Fig. 2a,b,c,f,g,i,j; Fig. 3a,b,e,f; Fig. 4b; Fig. 5c,d; Fig. 6a; Supplementary Figures S1a-h; S2b,c; S3a,b; S4a-d; S5a; S6; S7b; S8c; S9c).

Eukaryotic cell lines

Policy information about cell lines and Sex and Gender in Research

Cell line source(s)

HeLa (ATCC CCL-2), SKOV3 (ATCC HTB-77), MDA-MB-436 (ATCC HTB-130) and U2OS (ATCC HTB-96) cells were obtained from ATCC. RPE1 and 8988T cells were obtained from Dr. Alan D'Andrea (Dana-Farber Cancer Institute, Boston, MA). DLD-1 and DLD1-BRCA2KO cells (Horizon HD105-007) were obtained from Dr. Robert Brosh (National Institute on Aging, Baltimore, MD). HeLa-BRCA2KO cells were generated in our laboratory and previously described (Ref 65). To knock-out PARP10 and RAD18, commercially available CRISPR/Cas9 KO plasmids (Santa Cruz Biotechnology sc-406703 and sc-406099 respectively) were used. For re-expression of PARP10 wildtype and mutant variants, the pLV[Exp]-Puro-SV40>hPARP10 lentiviral constructs (Cyagen) were used.

Authentication

Authentication was performed regularly based on morphology and gene/protein expression (in case of genetic alterations)

Mycoplasma contamination

Cell lines tested negative for mycoplasma

Commonly misidentified lines<br>(See ICLAC register)	None of the cell lines used are listed on the ICLAC register version 10
